# Smoking during pregnancy in relation to grandchild birth weight and BMI trajectories

**DOI:** 10.1371/journal.pone.0179368

**Published:** 2017-07-12

**Authors:** Ming Ding, Changzheng Yuan, Audrey J. Gaskins, Alison E. Field, Stacey A. Missmer, Karin B. Michels, Frank Hu, Cuilin Zhang, Matthew W. Gillman, Jorge Chavarro

**Affiliations:** 1 Department of Nutrition, Harvard T.H. Chan School of Public Health, Boston, MA, United States of America; 2 Department of Epidemiology, Harvard T.H. Chan School of Public Health, Boston, MA, United States of America; 3 Division of Adolescent Medicine, Department of Medicine, Boston Children’s Hospital and Harvard Medical School, Boston, MA, United States of America; 4 Channing Laboratory, Department of Medicine, Brigham and Women’s Hospital and Harvard Medical School, Boston, MA, United States of America; 5 Division of Reproductive Endocrinology and Infertility, Department of Obstetrics, Gynecology, and Reproductive Biology, Brigham and Women's Hospital and Harvard Medical School, Boston, MA, United States of America; 6 Obstetrics and Gynecology Epidemiology Center, Department of Obstetrics, Gynecology and Reproductive Biology, Brigham and Women’s Hospital and Harvard Medical School Boston, MA, United States of America; 7 Epidemiology Branch, Division of Epidemiology, Statistics and Prevention Research, *Eunice Kennedy Shriver* National Institute of Child Health and Human Development, Bethesda, MD, United States of America; 8 Department of Population Medicine, Harvard Pilgrim HealthCare and Harvard Medical School, Boston, MA, United States of America; Universidade de Sao Paulo, BRAZIL

## Abstract

**Background:**

Maternal smoking has been linked to lower birth weight and higher risk of childhood obesity. However, it is unknown whether grand-maternal smoking during pregnancy is associated with grandchildren birth weight and body mass index (BMI) trajectories.

**Methods:**

We investigated associations of smoking during pregnancy with birth weight, risks of overweight and BMI trajectories among 46,858 mother-child dyads and 6,583 grandmother-mother-child triads of three cohort studies of related individuals. Smoking during pregnancy was reported by mothers, and anthropometric data were provided by participants in each cohort.

**Results:**

Compared to grandchildren of non-smoking women, grandchildren of women who smoked more than 14 cigarettes per day throughout pregnancy were 70 g (95% CI: 12, 129 g; P for trend = 0.01) heavier at birth, and 18% (95% CI: 4%, 34%; P for trend = 0.01) more likely to become overweight. The mean BMI of grandchildren of women who smoked during pregnancy was 0.45 kg/m^2^ (95% CI: 0.14, 0.75 kg/m^2^; P for trend = 0.006) higher through adolescence and young adulthood than that of grandchildren of non-smoking mothers.

**Conclusions:**

Grandmothers’ smoking during pregnancy was associated with higher birth weight, higher risk of overweight, and higher BMI through adolescence and young adulthood.

## Introduction

Poor fetal development has been increasingly recognized as a contributor to the incidence of non-communicable diseases, such as hypertension, type 2 diabetes, and cardiovascular disease [1,2]. Women born with low birth weight, a gross indicator of poor fetal growth, are more likely to have low birth weight infants [3], suggesting the possibility of transgenerational effect on health by insults during their own fetal development.

Seen as a fashionable behavior, the prevalence of smoking among women peaked in 1960s in U.S. [4,5]. Studies among women of this and subsequent generations helped establish the now well-recognized relationships of smoking during pregnancy with lower birth weight and with higher risk of obesity in both childhood and adulthood [6–12]. However, whether smoking during pregnancy could have transgenerational effects on the grandchildren’s birth weight and risk of obesity remains to be determined. Grandchildren of women who were smokers in the 1960s are today’s children, adolescents and young adults. Given that the prevalence of childhood and adolescent obesity was approximately 17% in 2009–2010 and childhood obesity is a strong risk factor of type 2 diabetes and cardiovascular disease in adulthood [13,14], it is of public health importance to examine whether the smoking epidemic 50 years ago is related to the current obesity epidemic in youth.

The goal of this study was to examine the associations of grand-maternal smoking during pregnancy with birth weight and risk of obesity in their grandchildren. To achieve this goal, we constructed a three-generation cohort by linking data from three cohorts of related individuals: the Nurses’ Mothers Cohort Study (NMCS), the ongoing Nurses’ Health Study II (NHS II) and the ongoing Growing Up Today Study 2 (GUTS2). The NMCS is comprised of mothers of women participating in NHSII and GUTS2 is comprised of offspring of women in NHS II, thus allowing us to study smoking effects across three generations.

## Methods

### Study population

NHS-II was established in 1989 when 116,430 female nurses (aged 25–42 years) completed a questionnaire about their lifestyle and medical history. Follow-up questionnaires are sent biennially to collect updated information. In 2001, 39,904 mothers of NHS-II participants completed a questionnaire regarding their pregnancy with their NHS-II participant daughter and additional information regarding their daughter’s infancy (Nurses’ Mothers Cohort Study). The Growing Up Today Study 2 (GUTS2) began in 2004 when 10,907 children (6,002 girls; 4,905 boys) age 9–14 years of women participating in NHS-II completed a baseline questionnaire regarding health and growth indicators. Follow-up questionnaires have been sent biennially to update information. Additional information on GUTS2 participants’ perinatal and early life exposures were collected in 2009 from 9,096 NHS-II participants with children enrolled in GUTS2. After excluding 1,652 individuals with missing information on maternal smoking status during pregnancy, we had data from 38,252 mother-offspring dyads between NMCS (F1) and the NHS-II (F2) cohorts, 8,606 mother-offspring dyads between the NHS-II (F2) and the GUTS2 (F3) cohorts, and 6,583 grandmother-mother-offspring triads (F1, F2, F3). The study was approved by the Institutional Review Boards of the Harvard T.H. Chan School of Public Health and Brigham and Women’s Hospital.

### Assessment of smoking

F1 (grandmother): NMCS (F1) participants reported on whether they had smoked during pregnancy, whether they had stopped smoking during pregnancy, and in which trimester they had stopped. NMCS (F1) participants provided further information on the number of cigarettes smoked per day. Smoking during pregnancy was categorized into ‘never smoked during pregnancy’, ‘only smoked during first two trimesters’, ‘smoked during all three trimesters, 1–14 cigarettes per day’, and ‘smoked during all three trimesters, ≥ 14 cigarettes per day’.

F2 (mother): NHS-II (F2) participants reported on whether they had smoked during pregnancy, whether they had stopped smoking during pregnancy, and in which trimester they had stopped. Smoking during pregnancy was categorized into ‘never smoked during pregnancy’, ‘only smoked during the first two trimesters’, and ‘smoked during three trimesters’.

A validation study conducted among 146 NMCS participants who had also participated in the National Collaborative Perinatal Project showed that the validity of recalled maternal smoking during pregnancy was high (sensitivity = 0.86, specificity = 0.94) [15].

### Assessment of anthropometric characteristics

F2 (mother): F2 participants reported their own height and weight at baseline, and current weight was updated on biennial questionnaires.

F3 (offspring): F2 participants reported the birth weight of F3 participants in 2009. The birth weight was recorded in 6 categories ranging from ≤5, 5.0–5.4, 5.5–6.9, 7–8.4, 8.5–9.9, and ≥10 lbs. Recalled offspring birth weight is shown to be of high validity and reproducibility from thirty or more years ago [15]. We created a continuous birth weight using the mean value of each category, and the birth weight for the 6 categories was 4.75, 5.25, 6.25, 7.75, 9.25, and 10.25, respectively. F3 participants reported their weight and height in 2004, 2006, 2008, and 2011.

Body mass index (BMI) was calculated as the ratio of weight (kg) to height (m) squared. In adolescents (< 18 y), overweight/obesity was defined as a BMI at or above the age- and sex-specific cutoffs proposed by the International Obesity Task Force (IOTF) [16]. In adults (≥ 18 y), overweight/obesity was defined as BMI ≥ 25 kg/m^2^. Self-reported height and weight by adolescents and adults are known to be sufficiently valid for use in epidemiologic research [17,18]. Once participants became overweight/obese they were considered overweight/obese for the remainder of the follow-up.

### Covariate assessment

We mailed NMCS (F1) participants questionnaires to report maternal age at the time of their daughters’ birth, gestational age, level of education, consumption of alcohol, vegetable, fruit, and meat, physical activity, and weight gain during pregnancy. All of the questions were asked using multiple choice response options, and we converted the categorical variables to continuous variables [19].

In F2 participants, social-economic status was measured by a question “how do you feel about your standing in US society using a ladder” in 2001. The ladder includes 10 levels from bottom to top, with a higher level indicating higher social-economic status. Dietary intakes were measured by 131-item food frequency questionnaire (FFQ), and we derived the AHEI-2010 score based on intake levels of 11 components, which were chosen on the basis of their association with chronic disease and mortality risk in observational and interventional studies [20]. The total score ranged from 0 (non-adherence) to 110 (perfect adherence), with a higher score indicating a higher quality of diet. Physical activity was measured by questionnaires, and we derived weekly energy expenditure in metabolic equivalent task-hours (MET-hours).[21]

### Statistical analysis

F1 smoking with F3 outcomes: Generalized estimating equation (GEE) regression models were used to evaluate the associations of F1 smoking with F3 birth weight and BMI trajectories from childhood through young adulthood accounting for both within-person and within-sibling correlation in outcomes. Log-binomial GEE regression models were used to examine the association of F1 smoking with F3 risk of overweight or obesity accounting for the non-independence of sibling clusters and confounding variables. We adjusted for gestational age, age at birth, level of education, consumption of alcohol, vegetable, fruit, and meat, physical activity, and weight gain during pregnancy. We additionally adjusted for F2 mothers’ pre-pregnancy BMI, smoking during pregnancy, social-economic status, and AHEI diet score and physical activity during pregnancy. We further plotted the F3 BMI trajectories according to F1 smoking status using a locally weighted scatterplot smoothing regression estimator with the LOESS function in R.

We conducted sensitivity analysis using time-varying overweight/obesity status assessed every other year as main outcome, and applied log-binomial GEE to examine the association between F1 smoking and F3 risk of overweight/obesity. To examine whether the association between grand maternal (F1-F3) smoking during pregnancy with offspring risk of overweight or obesity was mediated by F2 maternal smoking status, we restricted our analysis to F2 mothers who never smoked during pregnancy. To further evaluate whether the association between grand maternal (F1-F3) smoking during pregnancy with offspring risk of overweight or obesity was mediated by F2 mother pre-pregnancy BMI, we matched the F1 participants by F2 pre-pregnancy BMI, which was divided into < 20, 20, 21, 22, 23, 24–26, 26–28, ≥ 28 kg/m^2^ groups. We collapsed grand maternal smoking into ‘never smoked during pregnancy’, ‘smoked only during the first two trimesters or throughout pregnancy with 1–14 cigarettes per day’ and ‘smoked through three trimesters during pregnancy, ≥ 14 cigarettes per day’, and matched by a ratio of 7:1.5:1, which made the maximum use of the F1 participants.

All statistical analyses were performed using SAS Version 9.2 (SAS Institute Inc., Cary, NC, USA) and R 3.1.0.

## Results

The baseline characteristics according to grand-maternal smoking status during pregnancy are shown in Table 1. Among 38,252 NMCS participants (F1), 9,500 (24.8%) were smokers during pregnancy. Grand-maternal smoking during pregnancy (F1) was associated with higher prevalence of F2 maternal smoking during pregnancy. However, no associations of smoking with level of education, dietary intake, and physical activity were found in F1 grandmothers.

**Table 1:** Baseline characteristics according to grand-maternal smoking status during pregnancy.

	Never smoked during pregnancy	Smoked only during 1^st^ and 2^nd^ trimesters	Smoked during all three trimesters, 1–14 cigarettes/day	Smoked during all three trimesters, > 14 cigarettes/day
Number of Women	28,752	1,343	5,188	2,969
Gestational age (week)	38.3 ± 1.90	38.3 ± 2.1	38.1 ± 2.0	38.1 ± 2.0
Maternal age (year)	26.3 ± 0.4	25.3 ± 0.4	26.0 ± 0.4	25.7 ± 0.4
Pre-pregnancy BMI (kg/m^2^)	25.2 ± 3.6	25.0 ± 3.5	24.8 ± 3.3	25.1 ± 3.6
Level of education				
≤ 8 years (%)	5.5	2.5	2.3	2.4
High school (%)	61.0	55.9	56.8	58.9
College (%)	33.6	41.5	40.8	38.8
Caesarean section (%)	3.2	3.1	3.0	2.4
History of diabetes (%)*	10.7	8.8	8.5	10.5
Preeclampsia (%)	3.4	3.5	3.2	3.5
Prehypertension (%)	3.7	3.6	3.1	3.5
F2 maternal smoking during pregnancy (%)	3.3	4.8	4.1	6.6
*Food consumption and physical activity during pregnancy*				
Alcohol (g/day)	0.6 ± 1.9	1.3 ± 2.6	2.0 ± 4.2	3.0 ± 6.2
Vegetables (frequency/day)	2.0 ± 1.0	2.0 ± 1.1	1.9 ± 1.0	1.8 ± 0.9
Fruits (frequency/day)	1.6 ± 1.1	1.6 ± 1.1	1.5 ± 1.0	1.3 ± 0.9
Meat (frequency/day)	1.4 ± 0.6	1.5 ± 0.6	1.4 ± 0.5	1.5 ± 0.6
Outdoor activity (heavy labor, %)	3.1	2.0	2.3	2.9

*History of diabetes was assessed at the time of completing the questionnaire.

Continuous variables were presented as means.

As expected, maternal smoking during pregnancy was associated with lower offspring birth weight (P for trend < 0.001) (S1 Table). However, grand-maternal smoking was positively related to her grandchildren’s birth weight in a linear fashion. Compared to grandchildren of non-smoking women, grandchildren of women who smoked more than 14 cigarettes/day throughout pregnancy were 70g (95% CI: 12g, 129g) heavier at birth (p, linear trend = 0.01) (Table 2). This positive association persisted after further adjustment for F2 mothers’ pre-pregnancy BMI or smoking during pregnancy.

**Table 2:** Adjusted differences in offspring birth weight (g) according to grand-maternal smoking status.

	Never smoked during pregnancy	Smoked during the 1st and 2nd trimesters only	Smoked during all three trimesters, 1–14 cigarettes/day	Smoked during all three trimesters, > 14 cigarettes/day	P for trend
Participants	4,209	229	784	537	
Unadjusted model	Ref.	19 (-67, 106)	25 (-24, 74)	61 (5, 118)	0.03
Multivariate-adjusted model	Ref.	26 (-65, 116)	32 (-18, 83)	74 (12, 129)	0.01
Multivariate-adjusted model 1	Ref.	29 (-61, 119)	33 (-18, 83)	67 (9, 125)	0.02
Multivariate-adjusted model 2	Ref.	28 (-63, 118)	33 (-18, 83)	76 (18, 134)	0.009
Multivariate-adjusted model 3	Ref.	32 (-57, 121)	33 (-17, 83)	77 (18, 135)	0.008

Multivariate-adjusted model adjusted for gestational age (quartiles), age at birth (quartiles), level of education (≤ 8 years, high school, college), as well as consumptions of alcohol (continuous), vegetable (continuous), fruit (continuous), meat (continuous), physical activity (low, high), and weight gain (quartiles) during pregnancy.

Model 1 additionally adjusted for F2 mother pregnancy BMI (≤ 21, 21–23, 23–25, 25–30, 30–35, >35 kg/m^2^).

Model 2 additionally adjusted for F2 mother smoking during pregnancy (never smoker, smoked during the 1st and 2nd trimesters only, smoked during all three trimesters).

Model 3 additionally adjusted for F2 mother social-economic status (low, medium, high), F2 mother diet score (tertiles), and F2 mother physical activity (tertiles).

**Fig 1:**
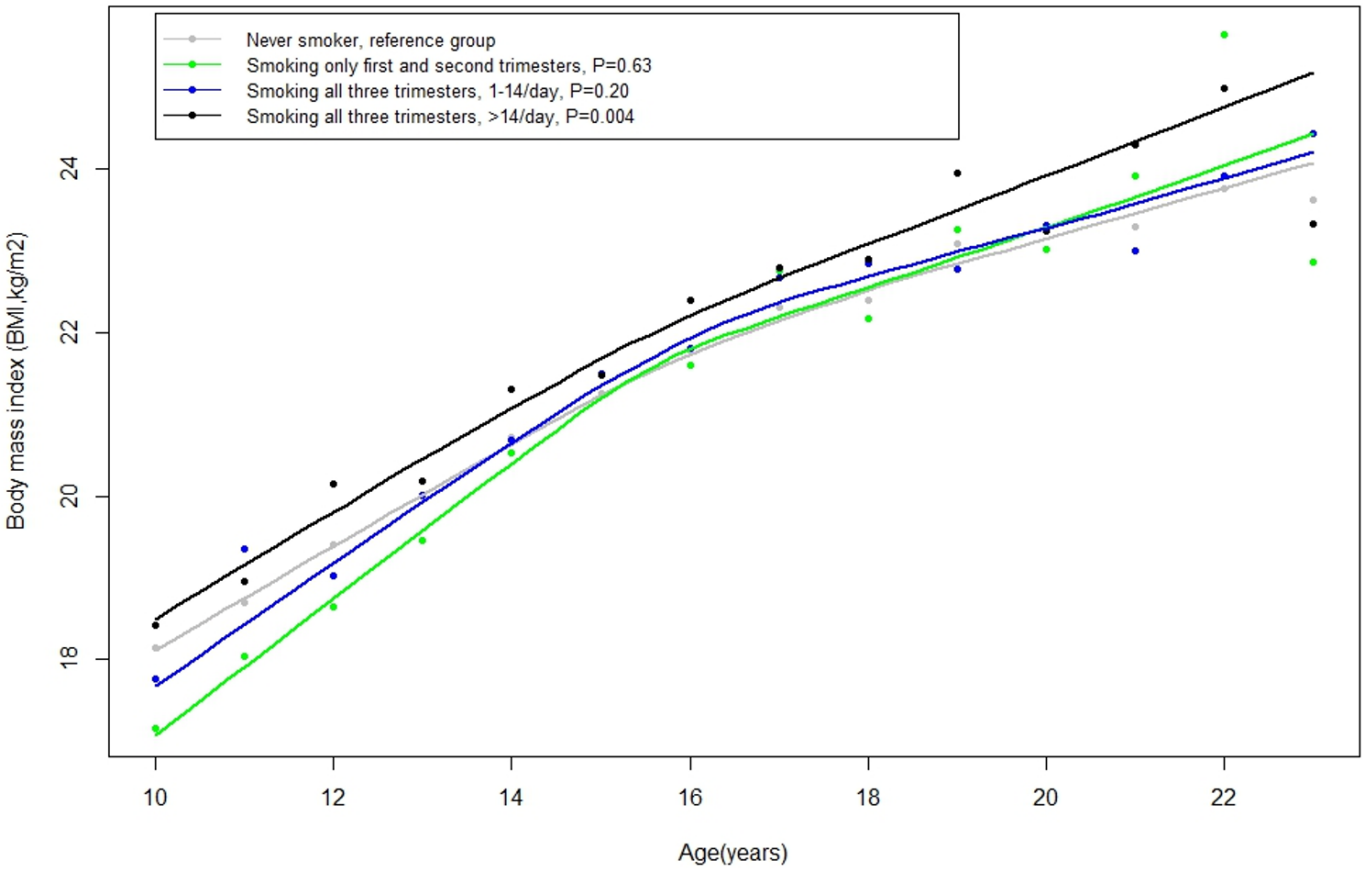
Grandmothers’ smoking during pregnancy and the BMI trajectory of the offspring (F1-F3). P values were obtained using generalized estimating equations (GEE) adjusting for age at BMI assessment (continuous), gestational age (quartiles), age at birth (quartiles), level of education (≤ 8 years, high school, college), as well as consumptions of alcohol (continuous), vegetable (continuous), fruit (continuous), meat (continuous), physical activity (low, high), and weight gain (quartiles) during pregnancy.

Grand-maternal smoking was positively related to grandchildren BMI. Compared to grandchildren of women who never smoked during pregnancy, grandchildren of women who smoked through the entire pregnancy had consistently higher BMI from ages 10 to 22 y (P = 0.01) (Fig 1). The difference in BMI trajectories was largest between grandchildren of non-smoking women and grandchildren of women who smoked more than 14 cigarettes per day through three trimesters of pregnancy (mean BMI difference: 0.45 kg/m^2^; 95% CI: 0.14, 0.75; P = 0.004) (Fig 1, Table 3). These differences in BMI trajectories translated into a higher frequency of overweight or obesity among grandchildren (Table 3). Specifically, the probability of ever becoming overweight or obese was 18% (95%CI: 4%, 34%) higher among grandchildren of women who smoked > 14 cigarettes/day throughout pregnancy when compared to grandchildren of non-smoking women. This association persisted after further adjusting for F2 maternal pre-pregnancy BMI, F2 maternal smoking during pregnancy, and F3 birth weight. The association remained positive when using time-varying overweight/obesity status as main outcome (S2 Table).

**Table 3:** Grand-maternal smoking status in relation to offspring BMI and risk of overweight/obesity.

	Never smoked during pregnancy	Smoked during the 1st and 2nd trimesters only	Smoked during all three trimesters, 1–14 cigarettes/day	Smoked during all three trimesters, > 14 cigarettes/day	P for trend
**BMI (kg/m**^**2**^)^*^					
Observations	13,999	743	2,616	1,707	
Unadjusted model	Ref.	-0.10 (-0.52, 0.32)	0.12 (-0.13, 0.37)	0.40 (0.10, 0.70)	0.01
Multivariate-adjusted model	Ref.	-0.10 (-0.51, 0.31)	0.16 (-0.09, 0.41)	0.45 (0.14, 0.75)	0.004
Multivariate-adjusted model 1	Ref.	-0.03 (-0.42, 0.35)	0.17 (-0.08, 0.42)	0.36 (0.07, 0.66)	0.01
Multivariate-adjusted model 2	Ref.	-0.09 (-0.50, 0.31)	0.14 (-0.11, 0.39)	0.41 (0.10, 0.72)	0.01
Multivariate-adjusted model 3	Ref.	-0.10 (-0.51, 0.30)	0.18 (-0.08, 0.43)	0.37 (0.05, 0.68)	0.02
**Risk of overweight/obesity**^&^					
Cases/participants	1,483/4,805	81/261	295/908	218/609	
Unadjusted model	1.00	1.01 (0.83, 1.23)	1.06 (0.95, 1.18)	1.16 (1.02, 1.31)	0.02
Multivariate-adjusted model	1.00	1.05 (0.86, 1.28)	1.07 (0.96, 1.20)	1.18 (1.04, 1.34)	0.01
Multivariate-adjusted model 1	1.00	1.07 (0.90, 1.28)	1.04 (0.93, 1.17)	1.13 (1.00, 1.28)	0.06
Multivariate-adjusted model 2	1.00	1.04 (0.86, 1.27)	1.07 (0.96, 1.20)	1.17 (1.03, 1.34)	0.01
Multivariate-adjusted model 3	1.00	1.03 (0.86, 1.25)	1.08 (0.96, 1.20)	1.12 (0.98, 1.28)	0.06

*Results for BMI were regression coefficients.

^**&**^ Results for overweight/obesity were relative risk.

Multivariate-adjusted model adjusted for gestational age (quartiles), age at birth (quartiles), level of education (≤ 8 years, high school, college), as well as consumptions of alcohol (continuous), vegetable (continuous), fruit (continuous), meat (continuous), physical activity (low, high), and weight gain (quartiles) during pregnancy.

Model 1 additionally adjusted for F2 mother pre-pregnancy BMI (≤ 21, 21–23, 23–25, 25–30, 30–35, >35 kg/m^2^).

Model 2 additionally adjusted for F2 mother smoking during pregnancy (never smoker, smoked during the 1st and 2nd trimesters only, smoked during all three trimesters).

Model 3 additionally adjusted for F2 mother social-economic status (low, medium, high), F2 mother diet score (tertiles), and F2 mother physical activity (tertiles).

We further examined the association of grand-maternal smoking with anthropometric outcomes in an analysis restricted to F2 mothers who never smoked during pregnancy. The positive associations with birth weight, risk of overweight, and BMI during follow-up persisted in this analysis (S3 Table). Moreover, the positive associations of grand-maternal smoking with birth weight and BMI persisted when we matched the F1 participants by F2 pre-pregnancy BMI (S4 Table).

## Discussion

Using an intergeneration study design, we found that grandmothers’ smoking during pregnancy was associated with higher birth weight, higher BMI, and higher risk of overweight in youth. These findings are significant in at least two aspects. First, they suggest that the high frequency of smoking in the 1960s may have contributed to fueling today’s high prevalence of childhood and adolescent obesity. If this relation holds to replication, it has important implications for the prevention of obesity and associated conditions over the following decades in developing regions of the world where smoking continues to increase, particularly among women of reproductive age [22]. Second, our study supports the more general hypothesis that exposures during pregnancy could impact health outcomes across multiple generations.

The literature on the relation of grandmaternal smoking during pregnancy and grandchild birth weight and BMI trajectories is scarce. Two studies have examined the association of grandmother smoking during pregnancy with birth weight of the offspring, and positive associations were only found when restricting to certain subpopulations [23,24]. A possible reason explaining the apparent discrepancy with our results is that grandmother smoking was only defined as yes and no, and this categorization may not have been fine enough to capture the association. In another previous study that examined the association of grandmothers’ smoking with obesity using data from the GUTS cohort there was a positive association that was restricted to girls at the age of 12 [25]. Nevertheless, the point estimates of the relation between grand-maternal smoking on risk of obesity between the previous publication and the current report are nearly identical suggesting that differences in study design, and specifically the choice of selecting a single grandchildren in the previous study rather than using all the data along with adequate statistical modeling of within-sibling correlations as done in the current report may have decreased the statistical power to identify associations in the previous report.

In our study, we found that grandmother smoking was associated with higher birth weight and higher BMI in the offspring, and the associations appeared to be independent of smoking and BMI during the second-generation pregnancy. Although the mechanism is not clear, the results of our study suggest that smoking may exert trans-generational effects on offspring birth weight and risk of overweight in the third generation. In fact, a transgenerational effect has been reported by other studies, due to adverse intrauterine environments. Specifically, in humans, low birth weight mothers were more likely to have low birth weight infants and men who were born after a pregnancy complicated by pre-eclampsia were more likely to father a first pregnancy affected by preeclampsia [3,26]. Animal studies have shown that undernutrition during pregnancy in mice reduced birth weight and increased impaired glucose tolerance and obesity in both the first and second generation offspring [27]. Further clarifying the nature of this relation may provide insights into the underlying biology of non-genetic trans-generational transmission of information and may also have clinical and public health implications.

The mechanism of smoking with obesity of the offspring has been investigated. One prospective study showed that infants exposed in utero to smoke had higher arterial cortisol and adrenocorticotropin hormone levels at birth [28]. Epigenome-wide association studies showed that maternal smoking during pregnancy was associated with differences in DNA methylation in newborns, and this association persisted until adolescence [29,30]. One study found no significant differences in methylation in newborns when compared according to their grandmother’s smoking habits while pregnant with the mother which might be due to demethylation and reprogramming in the formation of the zygote of the newborn [31]. Further studies are needed to confirm those findings and to clearly illustrate the mechanisms of the transgenerational effect of smoking during pregnancy.

The possibility that the associations are the result of unmeasured confounding cannot be excluded. First, grandmothers who were smokers during pregnancy might be from lower social-economic status (SES), and their offspring might be likely to be obese [32]. However, educational attainment, a marker of SES, was not strongly related to smoking in this study and grandmother’s smoking status during pregnancy remained associated with her grandchildren’s birth weight and BMI trajectories even after adjustment for grandmothers’ education, diet and physical activity. Nevertheless, there may still be residual confounding due to measurement error of the covariates which were collected retrospectively, although the measurement error might be independent of the F3 outcome. Second, the positive associations between F1 and F3 might be due to higher prevalence of smoking or higher BMI in F2 whose mothers smoked during pregnancy. However, we further adjusted for F2 smoking status and F2 BMI, and the positive associations did not change. Moreover, the positive associations also persisted when restricting to F2 mothers who were never smokers during pregnancy or matching F1 participants by F2 pre-pregnancy BMI. Third, there might be measurement error of the self-reported weight and height. However, measurement error of the outcome is non-differential with respect to the grand-maternal smoking status, and would not bias the effect estimates, although the confidence interval might be larger. Fourth, all of the F2 participants were nurses, representing a highly selected population. However, the prevalence of smoking during pregnancy in this cohort closely resembles the prevalence of smoking among women of reproductive age throughout the 1950s and 1960s in the United States [5], suggesting that the smoking rate of F1 mothers might still be representative for their generation. In fact, a highly selected population of F2 participants may have had the positive effect of partially controlling for SES, which may be a mediator between F1 smoking and F3 health outcomes.

In conclusion, our study showed that grandmothers’ smoking during pregnancy was associated with higher birth weight and higher risk of overweight from childhood through young adulthood. These findings point to a potential contributor of the current childhood obesity epidemic in the US. Furthermore, they may be informative of the effects on future generations in regions of the world where smoking among women of reproductive age remains high.

## Supporting information

S1 Table Adjusted differences in offspring birth weight (g) according to maternal smoking status. Model 1 adjusted for gestational age (quartiles), age at birth (quartiles), level of education (≤ 8 years, high school, college), as well as consumptions of alcohol (continuous), vegetable (continuous), fruit (continuous), meat (continuous), physical activity (low, high), and weight gain (quartiles) during pregnancy. Model 2 adjusted for F1 smoking status (never smoked during pregnancy, smoked during the 1st and 2nd trimesters only, smoked during all three trimesters with 1–14 cigarettes/day, smoked during all three trimesters with > 14 cigarettes/day), gestational age (< 38 weeks, 38–42 weeks, > 42 weeks), age at birth (quartiles), as well as alcohol consumption (continuous), aHEI (quartiles), physical activity (quartiles), and BMI before pregnancy (< 20.9 kg/m^2^, 21–22.9 kg/m^2^, 23–24.9 kg/m^2^, 25–29.9 kg/m^2^, 30–34.9 kg/m^2^, ≥ 35 kg/m^2^).
(DOCX)

S2 Table Grand-maternal smoking status in relation to risk of overweight/obesity using time-varying overweight/obesity status as main outcome. Multivariate-adjusted model adjusted for gestational age (quartiles), age at birth (quartiles), level of education (≤ 8 years, high school, college), as well as consumptions of alcohol (continuous), vegetable (continuous), fruit (continuous), meat (continuous), physical activity (low, high), and weight gain (quartiles) during pregnancy. Model 1 additionally adjusted for F2 mother pre-pregnancy BMI (≤ 21, 21–23, 23–25, 25–30, 30–35, >35 kg/m^2^). Model 2 additionally adjusted for F2 mother smoking during pregnancy (never smoker, smoked during the 1st and 2nd trimesters only, smoked during all three trimesters). Model 3 additionally adjusted for F2 mother social-economic status (low, medium, high), F2 mother diet score (tertiles), and F2 mother physical activity (tertiles).
(DOCX)

S3 Table Grand-maternal smoking status in relation to offspring BMI and risk of overweight/obesity restricting to F2 mothers who never smoked during pregnancy. *Results for birth weight and BMI were regression coefficients. ^**&**^ Results for overweight/obesity were relative risk. Multivariate model adjusted for gestational age (quartiles), age at birth (quartiles), level of education (≤8 years, high school, college), as well as consumptions of alcohol (continuous), vegetable (continuous), fruit (continuous), meat (continuous), physical activity (low, high), and weight gain (quartiles) during pregnancy.
(DOCX)

S4 Table Grand-maternal smoking status in relation to offspring BMI and risk of overweight/obesity by matching F2 pre-pregnancy BMI. *Results for birth weight and BMI were regression coefficients. ^**&**^Results for overweight/obesity were relative risk. Multivariate model adjusted for gestational age (quartiles), age at birth (quartiles), level of education (≤ 8 years, high school, college), as well as consumptions of alcohol (continuous), vegetable (continuous), fruit (continuous), meat (continuous), physical activity (low, high), and weight gain (quartiles) during pregnancy.
(DOCX)
