# Supplementary material for: Smoking during pregnancy in relation to grandchild birth weight and BMI trajectories
Source: PLoS One. 2017 Jul 12;12(7):e0179368. doi: 10.1371/journal.pone.0179368 (PMC5507479; doi:10.1371/journal.pone.0179368)
Supplement: S1 Table — Model 1 adjusted for gestational age (quartiles), age at birth (quartiles), level of education (≤ 8 years, high school, college), as well as consumptions of alcohol (continuous), vegetable (continuous), fruit (continuous), meat (continuous), physical activity (low, high), and weight gain (quartiles) during pregnancy. Model 2 adjusted for F1 smoking status (never smoked during pregnancy, smoked during the 1st and 2nd trimesters only, smoked during all three trimesters with 1–14 cigarettes/day, smoked during all three trimesters with > 14 cigarettes/day), gestational age (< 38 weeks, 38–42 weeks, > 42 weeks), age at birth (quartiles), as well as alcohol consumption (continuous), aHEI (quartiles), physical activity (quartiles), and BMI before pregnancy (< 20.9 kg/m2, 21–22.9 kg/m2, 23–24.9 kg/m2, 25–29.9 kg/m2, 30–34.9 kg/m2, ≥ 35 kg/m2). (DOCX) [file pone.0179368.s001.docx]

**S1 Table.**

|  | Never smoked during pregnancy | Smoked during the 1^st^ and 2^nd^ trimesters only | Smoked during all three trimesters | P for trend |  |
| --- | --- | --- | --- | --- | --- |
| **F1 smoking, F2 birthweight** | | |  |  |  |
| Unadjusted model | Ref. | -74 (-102, -46) | - 192 (-205, -180) | < 0.001 |  |
| Multivariate-adjusted model 1 | Ref. | -83 (-109, -57) | - 170 (-182, -157) | < 0.001 |  |
| **F2 smoking, F3 birthweight** | |  |  |  |  |
| Unadjusted model | Ref. | -168 (-272, -64) | -247 (-329, -165) | < 0.001 |  |
| Multivariate-adjusted model 2 | Ref. | -133 (-244, -23) | -283 (-362, -204) | < 0.001 |  |
| **Pooled** |  |  |  |  |  |
| Multivariate-adjusted model | Ref. | -86 (-111, -60) | -219 (-329, -110) | < 0.001 |  |
