# Supplementary material for: Smoking during pregnancy in relation to grandchild birth weight and BMI trajectories
Source: PLoS One. 2017 Jul 12;12(7):e0179368. doi: 10.1371/journal.pone.0179368 (PMC5507479; doi:10.1371/journal.pone.0179368)
Supplement: S2 Table — Multivariate-adjusted model adjusted for gestational age (quartiles), age at birth (quartiles), level of education (≤ 8 years, high school, college), as well as consumptions of alcohol (continuous), vegetable (continuous), fruit (continuous), meat (continuous), physical activity (low, high), and weight gain (quartiles) during pregnancy. Model 1 additionally adjusted for F2 mother pre-pregnancy BMI (≤ 21, 21–23, 23–25, 25–30, 30–35, >35 kg/m2). Model 2 additionally adjusted for F2 mother smoking during pregnancy (never smoker, smoked during the 1st and 2nd trimesters only, smoked during all three trimesters). Model 3 additionally adjusted for F2 mother social-economic status (low, medium, high), F2 mother diet score (tertiles), and F2 mother physical activity (tertiles). (DOCX) [file pone.0179368.s002.docx]

**S2 Table.**

|  | Never smoked during pregnancy | Smoked during the 1st and 2nd trimesters only | Smoked during all three trimesters, 1-14 cigarettes/day | Smoked during all three trimesters, > 14 cigarettes/day | P for trend |
| --- | --- | --- | --- | --- | --- |
| **Risk of overweight/obesity** |  |  |  |  |  |
| Cases/participants | 1,483/4,805 | 81/261 | 295/908 | 218/609 |  |
| Unadjusted model | 1.00 | 0.95 (0.78, 1.17) | 1.06 (0.95, 1.19) | 1.22 (1.07, 1.38) | 0.005 |
| Multivariate-adjusted model | 1.00 | 1.00 (0.81, 1.22) | 1.09 (0.96, 1.22) | 1.25 (1.10, 1.43) | 0.002 |
| Multivariate-adjusted model 1 | 1.00 | 1.03 (0.85, 1.25) | 1.06 (0.94, 1.19) | 1.19 (1.04, 1.36) | 0.02 |
| Multivariate-adjusted model 2 | 1.00 | 1.03 (0.85, 1.25) | 1.05 (0.93, 1.18) | 1.18 (1.03, 1.35) | 0.03 |
| Multivariate-adjusted model 3 | 1.00 | 1.03 (0.85, 1.24) | 1.05 (0.93, 1.18) | 1.18 (1.03, 1.34) | 0.03 |
