# Supplementary material for: Smoking during pregnancy in relation to grandchild birth weight and BMI trajectories
Source: PLoS One. 2017 Jul 12;12(7):e0179368. doi: 10.1371/journal.pone.0179368 (PMC5507479; doi:10.1371/journal.pone.0179368)
Supplement: S3 Table — *Results for birth weight and BMI were regression coefficients. & Results for overweight/obesity were relative risk. Multivariate model adjusted for gestational age (quartiles), age at birth (quartiles), level of education (≤8 years, high school, college), as well as consumptions of alcohol (continuous), vegetable (continuous), fruit (continuous), meat (continuous), physical activity (low, high), and weight gain (quartiles) during pregnancy. (DOCX) [file pone.0179368.s003.docx]

**S3 Table.**

|  | Never smoked during pregnancy | Smoked during the 1st and 2nd trimesters only | Smoked during all three trimesters, 1-14 cigarettes/day | Smoked during all three trimesters, > 14 cigarettes/day | P for trend |
| --- | --- | --- | --- | --- | --- |
| **Birth weight (g)*** |  |  |  |  |  |
| Participants | 3,769 | 199 | 699 | 444 |  |
| Unadjusted model | Ref. | 9 (-85, 103) | 10 (-42, 63) | 61 (0.3, 123) | 0.09 |
| Multivariate-adjusted model | Ref. | 22 (-77, 120) | 18 (-36, 72) | 69 (7, 132) | 0.049 |
| **BMI (kg/m^2^)*** |  |  |  |  |  |
| Observations | 12,505 | 663 | 2,247 | 1,419 |  |
| Unadjusted model | Ref. | -0.17 (-0.62, 0.27) | 0.07 (-0.20, 0.34) | 0.34 (0.02, 0.67) | 0.07 |
| Multivariate-adjusted model | Ref. | -0.17 (-0.59, 0.26) | 0.12 (-0.15, 0.39) | 0.44 (0.10, 0.77) | 0.02 |
| **Risk of overweight/obesity^&^** |  |  |  |  |  |
| Cases/participants | 1,266/4,106 | 66/219 | 239/748 | 169/482 |  |
| Unadjusted model | 1.00 | 0.98 (0.79, 1.22) | 1.04 (0.92, 1.18) | 1.13 (0.98, 1.30) | 0.10 |
| Multivariate-adjusted model | 1.00 | 1.03 (0.83, 1.29) | 1.07 (0.94, 1.21) | 1.18 (1.02, 1.37) | 0.03 |
