# Supplementary material for: Smoking during pregnancy in relation to grandchild birth weight and BMI trajectories
Source: PLoS One. 2017 Jul 12;12(7):e0179368. doi: 10.1371/journal.pone.0179368 (PMC5507479; doi:10.1371/journal.pone.0179368)
Supplement: S4 Table — *Results for birth weight and BMI were regression coefficients. &Results for overweight/obesity were relative risk. Multivariate model adjusted for gestational age (quartiles), age at birth (quartiles), level of education (≤ 8 years, high school, college), as well as consumptions of alcohol (continuous), vegetable (continuous), fruit (continuous), meat (continuous), physical activity (low, high), and weight gain (quartiles) during pregnancy. (DOCX) [file pone.0179368.s004.docx]

**S4 Table.**

|  | Never smoked during pregnancy | Smoked during 1^st^ and 2^nd^ trimesters only or during all three trimesters 1-14 cigarettes/day | Smoked during all three trimesters > 14 cigarettes /day | P for trend |
| --- | --- | --- | --- | --- |
| **Birth weight (g)*** |  |  |  |  |
| Participants | 3,045 | 638 | 434 |  |
| Unadjusted model | Ref. | -0.30 (-54.91, 54.30) | 63.88 (-0.61, 128.37) | 0.10 |
| Multivariate-adjusted model | Ref. | 9.15 (-47.77, 66.06) | 74.95 (7.36, 142.54) | 0.049 |
| **BMI (kg/m^2^)*** |  |  |  |  |
| Observations | 10,087 | 2,068 | 1,400 |  |
| Unadjusted model | Ref. | -0.08 (-0.35, 0.19) | 0.25 (-0.08, 0.58) | 0.30 |
| Multivariate-adjusted model | Ref. | 0.02 (-0.26, 0.29) | 0.35 (0.01, 0.69) | 0.08 |
| **Risk of overweight/obesity^&^** |  |  |  |  |
| Cases/participants | 1,083/3,472 | 225/734 | 170/496 |  |
| Unadjusted model | 1.00 | 0.99 (0.87, 1.12) | 1.11 (0.96, 1.27) | 0.27 |
| Multivariate-adjusted model | 1.00 | 1.01 (0.89, 1.15) | 1.15 (0.99, 1.33) | 0.11 |
